# Peripartal Rumen-Protected L-Carnitine Manipulates the Productive and Blood Metabolic Responses in High-Producing Holstein Dairy Cows

**DOI:** 10.3389/fvets.2021.769837

**Published:** 2021-12-24

**Authors:** Mohsen Danesh Mesgaran, Hassan Kargar, Sadjad Danesh Mesgaran, Ali Javadmanesh

**Affiliations:** ^1^Department of Animal Science, Faculty of Agriculture, Ferdowsi University of Mashhad, Mashhad, Iran; ^2^Kaesler Nutrition GmbH, Cuxhaven, Germany; ^3^Stem Cell Biology and Regenerative Medicine Research Group, Research Institute of Biotechnology, Ferdowsi University of Mashhad, Mashhad, Iran

**Keywords:** L-carnitine, cow, periparturient period, milk, metabolism

## Abstract

This study aimed to monitor the effect of including rumen-protected L-carnitine (Carneon 20 Rumin-Pro, Kaesler Nutrition GmbH, Cuxhaven, Germany) in the transition diet on the productive and metabolic responses of multiparous high-producing Holstein dairy cows. Thirty-two multiparous cows were allocated in a completely randomized design to receive the same diet plus 60 g fat prill containing 85% palmitic acid (control, *n* = 16) or 100 g rumen-protected L-carnitine (RLC, *n* = 16); at 28 days before expected calving until 28 days in milk (DIM). Fat prill was included in the control diet to balance the palmitic acid content of both experimental diets. Milk production over the 28 DIM for the control and RLC groups was 46.5 and 47.7 kg, respectively. Milk fat content tended to increase upon rumen-protected L-carnitine inclusion (*p* = 0.1). Cows fed rumen-protected L-carnitine had higher fat- and energy-corrected milk compared with the control group. Pre- and post-partum administration of L-carnitine decreased both high- and low-density lipoprotein concentrations in peripheral blood of post-partum cows. The results of this study indicated that the concentration of triglycerides and beta-hydroxybutyrate was not significantly different between the groups, whereas the blood non-esterified fatty acid concentration was markedly decreased in cows supplemented with L-carnitine. Animals in the RLC group had a significant (*p* < 0.05) lower blood haptoglobin concentration at 7 and 14 DIM than the control. Animals in the RLC group had a lower concentration of blood enzymes than those of the control group. The mRNA abundance of Toll-like receptors 4, cluster of differentiation 14, and myeloid differential protein 2 did not significantly change upon the supplementation of L-carnitine in the transition diet. In summary, the dietary inclusion of RLC improved dairy cow's performance during the early lactation period. Greater production, at least in part, is driven by improved energy utilization efficiency and enhanced metabolic status in animals during the periparturient period.

## Introduction

In dairy cows, the transition from gestation to lactation is challenged by energy requirement for milk production and secretion, inadequate feed intake, and metabolic disorders (1, 2). Therefore, this period is critical for determining the productive responses, metabolic health (3), and profitability of the dairy cows (4). As parturition approaches, concentrations of various hormones and metabolites begin to alter in order to support the milk yield (2). This would eventually lead to higher milk production while there is a lag in the dry matter intake (DMI) to provide nutrient demands of the animals post-partum. This phenomenon triggers the animals to mobilize the body fat reservoirs, which enters them in a state of negative energy balance and could last for a long period (i.e., several months) in various cases (5). A severe negative lag between DMI and milk yield is a risk factor for metabolic imbalancing as well as infectious and reproductive disorders (6). Besides, metabolic imbalancing initiates a cluster of risk factors in dairy cows which leads to an increased susceptibility to certain health disorders (2, 7). For example, fresh cows, which cannot meet energy demands through DMI, are associated with higher blood biomarkers of fat mobilization, such as non-esterified fatty acids (NEFA) (7, 8). Excessive fat mobilization may result in overproduction of the ketones, e.g., beta-hydroxybutyrate (BHB) (9, 10). This can be further elaborated by a higher degree of mobilization of energy reserves due to the severe negative energy balance and lower rumen fill index because of decreased DMI. Hence, the body condition score (BCS) may be considered as an indicator for the mobilization of adipose tissue. In particular, the post-partum decrease in BCS is associated with metabolic imbalancing and infectious disorders (11). Cows with a metabolic challenge have a more pronounced decrease in BCS from days 14 to 35 after calving, indicative of a higher degree of body fat mobilization.

Previous works have clearly pinpointed the state of immune dysregulation in dairy cows during the transition period (12, 13). The presence of any metabolic imbalancing resulted from aberrant nutrient metabolism, causing metabolic stress and inflammatory responses in the early lactating cows (1). Higher concentrations of NEFA and BHB may influence early lactation disease and alter immune competence (14, 15). Pathological levels of both NEFA and BHB have been negatively associated with polymorphonuclear leukocytes and peripheral blood mononuclear cell functionality (16–18). In particular, increasing levels of NEFA during the periparturient period manipulate the inflammatory response of dairy cows *via* its impact on Toll-like receptors (TLR) and their signaling pathways (19). TLR are among the pathogen recognition receptors within the immune system. Toll-like receptors 4 (TLR4) are able to recognize lipopolysaccharides (LPS), i.e., endotoxins of gram-negative bacteria, which are located in various cell types (20). Cluster of differentiation 14 (CD14) are accessory proteins, which facilitate the interaction between LPS and TLR4. Subsequently, CD14 transfers LPS to myeloid differential protein 2 (MD2), a protein complexed with TLR4 on the cell surface, which initiates the myeloid differentiation factor 88 pathway and in turn transcription of the inflammatory cytokines.

Carnitine is a water-soluble quaternary amine, which influences the function of all living cells. In dairy cows, L-carnitine is a necessary molecule for the normal activity of the tissues encompassing mitochondria. This molecule is involved in the shuttle of activated long-chain fatty acids from cytosol to the mitochondria. Besides, this molecule has a potential to influence, although indirectly, the rate of energy production from glucose (21, 22). In addition, previous experiments in monogastrics have demonstrated that L-carnitine has an antioxidative potential, which is quite vital for scavenging the excess reactive oxygen species and maintaining the health of livestock (23). L-Carnitine has been shown to modulate the liver inflammation as well as circulating pro-inflammatory markers *via* specific signaling pathways (24). However, in a recent study in mid-lactating dairy cows challenged with LPS, authors were not able to reveal significant effects of L-carnitine supplementation before and 2 weeks after the challenge (25). The aforementioned functions indicate that L-carnitine may play a unique function in the transition period. Therefore, the present work aimed to investigate the influence of dietary inclusion of L-carnitine, through a rumen-protected L-carnitine (Carneon 20 Rumin-Pro containing 20% L-carnitine, Kaesler Nutrition GmbH, Cuxhaven, Germany) product, during the transition period on (a) milk yield, chemical composition, and fatty acid profile; (b) plasma concentrations of glucose, urea, albumin, cholesterol, high-density lipoprotein (HDL), low-density lipoprotein (LDL), triglyceride, haptoglobin, calcium, blood enzymes, i.e., alanine aminotransferase (SGPT) and aspartate aminotransferase (SGOT), NEFA, and BHB; (c) mRNA expression of CD14, TLR4, and MD2; and (d) distinct animal behavioral indices such as rumen fill index, manure score, rumination activity, and BCS. Determining the aforementioned parameters would enable to observe the impact of rumen-protected L-carnitine on the productivity of the animal as well as their inflammatory status at the molecular level.

## Materials and Methods

### Animals, Feeding, and Management

Thirty-two multiparous Holstein cows (average 305-day milk yield of 12,000 kg in the previous lactation) were paired by expected calving date and randomly assigned to receive a similar basal diet plus 60 g fat prill containing 85% palmitic acid (control group, *n* = 16) or 100 g of the rumen-protected L-carnitine (RLC group, *n* = 16), beginning at 28 days before the expected calving through 28 days in milk (DIM). The rumen-protected L-carnitine top-dressed on the basal diet was Carneon 20 Rumin-Pro (Kaesler Nutrition GmbH, Cuxhaven, Germany), which is a commercial source of L-carnitine (20% L-carnitine coated with rumen bypass fat rich in palmitic acid). As described previously (21), the specific inclusion rate of a protected fat, i.e., fat prill, was included in the control group in order to balance the coated fat of the protected L-carnitine source. Hence, the experimental diets were either without or with extra supplemented L-carnitine through the rumen-protected L-carnitine source. The specific inclusion rate of protected L-carnitine source was decided based on the previous research observing the impact of L-carnitine around parturition and during the high lactation period (21, 26). During pre- and post-partum, the cows were housed in two separated free stall barns, while they had free access to feed and water in a commercial dairy farm with 820 milking cows. Diets were fed as a total mixed ration (TMR) two times per day at 0730 to 1830 h in amounts that ensured *ad libitum* consumption and ~4–8% feed refusals. The ingredients and chemical composition of the pre- and post-partum diets are presented in Table 1. Daily samples of TMR and feed refusal and weekly samples of the diet ingredients were collected, dried in a forced air oven for 72 h at 55°C, and ground using a Wiley mill to pass a 1-mm screen, then analyzed for DM and the chemical composition (27). Dry matter was determined after 24 h at 95°C (ISO 6496). Ash was determined after 3 h at 550°C (ISO 5984). Nitrogen was assessed using the Kjeldahl method (Kjeltec 2300 Autoanalyzer, Foss Tecator AB, Hoganas, Sweden) with crude protein (CP) as N × 6.25. Starch content was evaluated by an anthrone/sulfuric acid method using glucose as standard and estimated as 0.9 × glucose content after liberating the starch by heating in a boiling water bath in the presence of 2 N HCl (28). For NDF and ADF, the method of Goering and Van Soest (29) was used.

**Table 1:** Ingredient, chemical composition, and calculated energy content of pre-partum (from −28 to parturition) and post-partum (from 1 to 28 DIM).

**Items**	**Pre-partum**	**Post-partum**
Ingredient, % of DM		
Corn silage	35.0	17.6
Alfalfa hay	17.2	21.8
Wheat straw	4.8	0.6
Corn grain	8.6	12.3
Barley grain	7.9	14.1
Wheat grain	7.3	-
Sugar beet pulp	1.2	2.6
Extuded soybean meal	4.8	10.9
Wheat bran	1.3	6.3
Cottonseed whole	2.6	3.9
DDGS	1.3	2.7
Rape seed meal	1.3	3.2
Supplement^a^, ^b^	6.6	3.9
Metabolizable energy (MJ/kg DM) Chemical composition (% DM) and	10.3	11.2
CP	11.8	15.8
NDF	39.5	36.3
ADF	21.9	18.0
ASH	7.8	7.3
Starch + soluble sugar	25.3	27.3
EE	3.1	4.0

a *Pre-partum: contained 400 g anionic salts (www.javanehkhorasan.com), 200 g VitalG (rumen protected glucose, www.groupsana.comena), 100 g OptiMate (essential omega-3 from salmon oil, rumen protected with vitamins, www.agritech.ie), 15 g encapsulated choline chloride (www.Kemin.com), 25 g Lutrell^®^ Pure [conjugated linoleic acid (CLA), BASF], 60 g vitamin D3 (5,000,000 iu/kg), 80 g vitamin E and Se (11,000 iu and 300 mg/kg, respectively), 200 g mineral/vitamin premix/kg (vitamins including A: 1,500,000 iu, D3: 400,000 IU, E:3,000 IU, biotin: 120 mg; minerals including Ca, P, Mg Na, K, Mn Zn, Cu, Se, I, Fe, Co, and S with the quantity of 44, 20, 2.3, 20, 1.6, 3.4, 6, 5, 0.14, 0.25, 4 0.043, and 17.6 g, respectively, www.javanehkhorasan.com), and 15 g toxin bonder (Toxytrap, Iran).*

b *Post-partum: contained 160 g sodium bicarbonate (Petro Tarh, Iran), 60 g di-calcium phosphate (www.javanehkhorasan.com), 200 g VitalG (Rumen protected glucose, www.groupsana.comena), 150 g Optimate (essential omega-3 from salmon oil, rumen protected with vitamins, www.agritech.ie), 30 g encapsulated choline chloride (www.Kemin.com), 25 g Lutrell^®^ Pure [conjugated linoleic acid (CLA), BASF], 60 g vitamin D3 (5,000,000 iu/kg), 80 g vitamin E and Se (11,000 iu and 300 mg/kg, respectively), 200 g mineral/vitamin premix/kg (vitamins including A: 1,500,000 iu, D3: 400,000 IU, E:3,000 IU, biotin: 120 mg; minerals including Ca, P, Mg Na, K, Mn Zn, Cu, Se, I, Fe, Co, and S with the quantity of 44, 20, 2.3, 20, 1.6, 3.4, 6, 5, 0.14, 0.25, 40.043, and 17.6 g, respectively, www.javanehkhorasan.com), and 15 g Toxin bonder (Toxytrap, Iran)*.

Cows were milked three times daily at ~0400, 1200, and 2000 h. The incidence of health problems was accurately recorded for each cow throughout the experiment.

### Sample Collection and Processing

Feed refusals of each group were measured daily, and feed intake for each group was determined by difference assuming a different DM content of feed offered and the ort. Milk yield was recorded daily but reported from 3 days after calving. Weekly milk samples, for 4 weeks after the calving, from individual cows were obtained at 3 consecutive milking, preserved with 2-bromo-2-nitropropane-1,3-diol, and analyzed for protein, fat, lactose, milk urea nitrogen (MUN), somatic cell scores (SCC), solid non-fat (SNF), and total solid content using Fourier-transform infrared spectroscopy (FT-IR; CombiScope FTIR 600 HP, Delta Instruments, Drachten, The Netherlands) in a commercial laboratory (Sazan Rojan Alvand Co., Alborz, Iran). In addition, the milk fat samples obtained from cows at 21 DIM were analyzed for fatty acid composition. This particular sampling day for milk fatty acid composition analysis was chosen as feed intake in animals around 21–28 days in lactation would be higher (30); hence, the milk fatty composition would be more heavily relied from the diet rather than mobilized fat depot (31). For this, fatty acid methyl esters were prepared by transmethylation and were then quantified by using a gas chromatograph (Shimadzu GC-2010, Kyoto, Japan) equipped with a flame-ionization detector and a CP-7489 fused-silica capillary column (100 m × 0.25 mm i.d. with 0.2-μm film thickness; Varian, Walnut Creek, CA, USA). The initial oven temperature (50°C) was held for 1 min then ramped at 5°C/min to 160°C, where it was held for 42 min and then ramped at 5°C/min to 190°C and held for 22 min. Inlet and detector temperatures were maintained at 250°C, and the split ratio was 100:1. The hydrogen carrier gas flow rate through the column was 1 ml/min. The hydrogen flow to the detector was 30 ml/min, airflow was 400 ml/min, and the nitrogen make-up gas flow was 25 ml/min. Peaks in the chromatogram were identified and quantified using pure methyl ester standards.

Fat-corrected milk standardized to 4% fat was calculated using the equation of Gaines (32), FCM = [0.4 × milk yield (kg)] + [15 × milk fat (kg)], and ECM was calculated as presented by Muñoz et al. (33).

Bleeding was conducted from 10 cows per each group at 0800 h *via* puncture of the coccygeal vessels on days −14, −7, +7, +14, and +21 relative to calving as proposed by Greenfield et al. (34). The aforementioned sampling dates were chosen, as previous studies have shown extensive metabolic changes from 2 weeks pre- until 2 weeks post-partum, which could be associated with overall health alteration in dairy cows and higher culling rates (35). Samples on day 21 after calving was also taken to ensure a better depiction of dynamics of the selected metabolic and health parameters. The samples were kept at room temperature, and the serum was separated within 0.5 h, then stored frozen at −20°C until analyses for glucose (GOD-PAP, https://parsazmun.de/GLUCOSE/), triglycerides (GPO-POD, www.Bionik.web.com), NEFA (colorimetric method, Randox, County Antrim, UK), BHB (kinetic enzymatic method, Randox, County Antrim, UK), urea (http://paadco.co), cholesterol (CHOD_POG, http://paadco.co), HDL (direct enzymatic colorimetric method, http://paadco.co), LDL (direct enzymatic colorimetric method, http://paadco.co), SGOT (kinetic UV method based on IFCC recommendations, http://paadco.co), SGPT (kinetic UV method based on IFCC recommendations, http://paadco.co), calcium (Arsenazo III Colorimetric method, http://paadco.co), albumin (BGC method, https://parsazmun.de), and haptoglobin (an immunoturbidimetric assay).

### RNA Isolation, Reverse Transcription, and Quantitative Real-Time PCR

Samples of blood were obtained at 0800 h *via* puncture of the coccygeal vessels on −7, +7, and +14 days related to calving. These sampling points were chosen, as previous studies revealed that unresolved subacute inflammations as early as 7 days post-calving would damage the productivity of dairy cows in the subsequent lactation (36). These samples were immediately frozen at −80°C and used for the analysis of mRNA expression. Total RNA was extracted by the AccuZol™ Total RNA Extraction Solution (Bioneer, Daejeon, South Korea) according to the manufacturer's instruction. The purity and integrity of RNA were assessed using the Epoch microplate spectrophotometer (BioTek, Winooski, USA) and agarose gel electrophoresis, respectively. One μg of RNA was treated with DNase and reverse transcribed to cDNA using AccuPower^®^ RT PreMix (Bioneer, Daejeon, South Korea) according to the supplier's instruction.

The cDNA was then subjected to real-time quantitative PCR (qPCR) for amplification. Oligonucleotide primers specific for studied and reference genes were used (Table 2). All qPCR reaction conditions were in compliance with MIQE [minimum information for publication of qPCR experiments, (39)]. Quantitative PCR was performed in duplicate, using the RealQ Plus 2X master mix (Ampliqon, Odense, Denmark) in a LightCycler^®^ 96 instrument (Life Technologies Roche Life Science, Basel, Switzerland). Amplification was performed in 0.1-ml 8-strip tubes (Gunster Biotech, Viluppuram, Taiwan) as the reaction mixture containing 2 μl of cDNA, 5 pmol of each primer, and 10 μl of 2 × master mix in a total volume of 20 μl. The following PCR program was used: the initial step of 95°C for 10 min and the amplification step of 40 cycles which started with 15 s at 95°C followed by 30 s at 60°C and 20 s at 72°C. This program was followed by analyzing the melting curve performed with linear heating from 60 to 90°C. Reaction efficiency was calculated based on the slope of the standard curve (equation: efficiency = (10 (−1/slope) – 1) × 100). Correlation coefficients (*R*^2^ ≥ 0.99) were considered. The relative copy number of CD14, TLR4, and MD2 transcripts was normalized to the geometric means of both RPS9 and GAPDH reference genes.

**Table 2:** Species-specific primers for the quantification of selected as well as reference genes using a real-time qPCR assay.

**Name**	**Sequence**	**References**	**Product size (bp)**
TLR4-F^a^	CCTTGCGTACAGGTTGTTCC	(37)	129
TLR4-R^b^	GCCTAAATGTCTCAGGTAGTTAAAGC		
CD14-F	CACCACATTGCACACCTGTT	(37)	124
CD14-R	CACCACATTGCACACCTGTT		
MD2-F	GGAGAATCGTTGGGTCTGCT	(37)	92
MD2-R	GCTCAGAACGTATTGAAACAGGA		
GAPDH-F	TCATTGAAGCCTTCACTACATGGTCT	(37)	147
GAPDH-R	TGATGTTGGCAGGATCTCG		
RPS9-F	TAGGCGCAGACGGGCAAACA	(38)	136
RPS9-R	CCCATACTCGCCGATCAGCTTCA		

a *F: forward primer.*

b *R: reverse primer*.

### Animal Behavior

The weekly animal behavior including the time spent ruminating was recorded every 10 min per 24 h and calculated by multiplying the total number of observed activities in each duration (40). The body condition score, manure score, and rumen fill score were assessed weekly from 21 days before the expected parturition to 4 weeks after calving. The body condition score was recorded by the same operator using a 1–5 scale with 0.1 intervals as proposed by Ferguson et al. (41) and Roche et al. (11). Both the manure score (42) and rumen fill score (43) were assessed 6 times per day which started 2 h before the morning feeding by the same operator using a 1–5 scale.

### Statistical Analysis

Data obtained weekly were statistically analyzed using the Proc Mixed procedure of SAS (44) for a completely randomized design with repeated measures. The model included the effects of group, day relative to calving, and the interaction between group and day. Days relative to calving was used as a repeated measurement, with cow within experimental groups as the subject. Daily DMI of the groups was analyzed as previously described (45); however, the interaction between group and day was taken out from the model. A gamma-type function model (Y = a^*^EXP – cd) was generated to describe the relation between daily milk production (Y) and time (d), while (c) is the slope. Data of milk fatty acid composition were analyzed using a completely randomized design. Data of gene expression were analyzed in JMP^®^ 4.0 (SAS Institute, Cary, NC, USA) using the analysis of variance method (ANOVA) by least-square fit. Differences were considered significant at *p* < 0.05, whereas tendency was determined at 0.05 < *p* < 0.1. Data are expressed as the mean ± SEM.

## Results

### Milk Production and Composition

**Figure 1:**
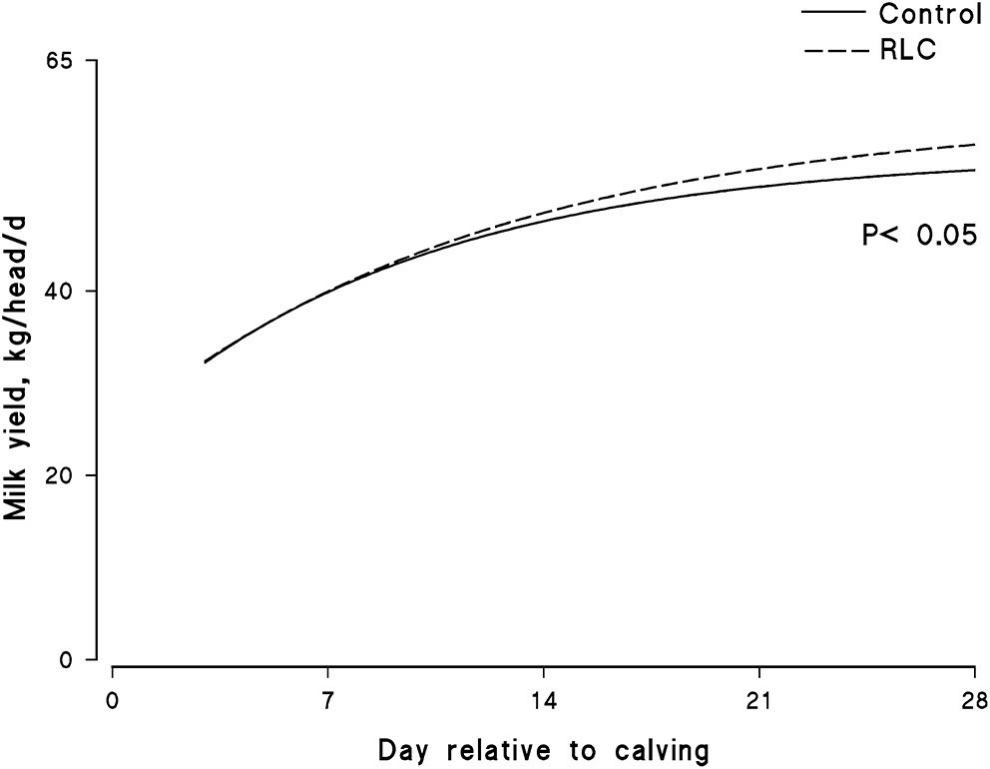
Effects of supplementing multiparous Holstein cows during the transition period with rumen-protected L-carnitine (Carneon 20 Rumin-pro, Kaesler Nutrition GmbH, Cuxhaven, Germany) on lactation curve of early-lactating Holstein dairy cows. A gamma-type function model (Y = a*EXP –cd) was generated in order to describe the relation between daily milk production (Y) and time (d), while (c) is the slope in Holstein dairy cows during the transition period. The group fed rumen-protected L-carnitine depicted as RLC.

Dry matter intake and the productive responses of the cows within the experimental groups are depicted in Table 3. There was no significant difference in feed intake between groups during this study (21.77 vs. 22.15 kg for the control and RLC group, respectively). The mean milk production over the 28 DIM for the control and LC group was 46.5 and 47.7 kg, respectively. The mathematical model indicated that the rate of increase in the milk production of the animals in the RLC group, compared with the control, was maximum after 10–15 days post-calving (Figure 1). The milk component content and production of the cows over the 4-week study period are presented in Table 3. Overall, milk fat content showed a tendency to increase by 10% upon rumen-protected L-carnitine supplementation in the transition diets (*p* = 0.1). There was no group or group-by-day interaction effect on the content of milk protein or lactose concentration. However, there was a significant effect of group and day (*p* < 0.05) on milk protein yield (1.48 vs. 1.51 g/day for the control and RLC group, respectively). Both 4% fat-corrected milk and energy-corrected milk were significantly (*p* < 0.05) influenced by the experimental group and DIM. Cows in the RLC group had roughly 4 kg higher fat- and energy-corrected milk compared with those of the control group. Milk urea-N content did not show any significant differences between the experimental groups. Milk SCC clearly decreased (*p* < 0.05) in the cows fed the transition diet supplemented with the rumen-protected L-carnitine.

**Table 3:** Milk production and composition of lactating Holstein dairy cows fed a post-partum diet plus fat prill containing 85% palmitic acid (control group) or Carneon 20 Rumin-pro (RLC group).

**Items**	**Experimental groups**								**SEM**	***p-value***		
	**Control**				**RLC**					**Group**	**Time**	Group^*^time
	**+7 days**	**+14 days**	**+21 days**	**+28 days**	**+7 days**	**+14 days**	**+21 days**	**+28 days**				
DMI (kg)	15.9	20.2	24.1	26.9	16.1	20.9	24.4	27.2	1.71	ns	^*^	-
Milk yield (kg)	36.2	44.7	50.1	52.3	36.0	45.0	52.1	54.5	1.01	ns	^*^	ns
Fat (%)	4.16	3.28	3.07	3.26	4.12	3.59	3.90	3.65	0.22	0.10	^*^	ns
Protein (%)	3.80	3.20	3.10	3.00	3.80	3.20	3.10	3.00	0.06	ns	^*^	ns
Lactose (%)	4.10	4.50	4.50	4.50	4.20	4.40	4.50	4.50	0.03	ns	ns	ns
Solids (%)	13.2	12.1	11.8	11.9	13.1	12.3	12.5	12.2	0.24	^*^	^*^	ns
SNF (%)	9.00	8.80	8.70	8.60	9.00	8.70	8.60	8.50	0.08	^*^	^*^	ns
4% FCM (kg)	37.1	39.8	43.1	46.5	36.7	42.2	51.4	51.6	0.91	^*^	^*^	ns
ECM (kg)	41.3	43.9	47.5	50.5	40.9	46.0	55.0	55.3	1.05	^*^	^*^	ns
Fat (g/day)	1.51	1.47	1.54	1.70	1.48	1.62	2.03	1.99	0.05	^*^	^*^	ns
Protein (g/day)	1.38	1.43	1.55	1.57	1.37	1.44	1.62	1.63	0.01	^*^	^*^	ns
MUN (g/dL)	12.1	14.0	14.0	13.5	13.3	13.5	12.8	13.2	0.43	ns	ns	ns
SCC (×1,000)	305	397	117	146	85.3	108	113	137	42.0	^*^	^*^	ns

*d: day relative to calving;*

* *p < 0.05; ns: p > 0.05; when the difference between means is >2 times the SEM, it is considered as significant (p < 0.05)*.

Table 4 depicts the effect of the experimental groups on milk fatty acid profiles. In the current study, cows in both groups had generally similar milk fatty acid concentrations. Nevertheless, cows in the RLC group had significant lower concentrations of C14:1 cis-9, C15:0, C16:1 cis-9, and C18:2 and higher C18:1 trans-9, trans-11 content in comparison with the control (*p* < 0.05).

**Table 4:** Milk fatty acid profile of lactating Holstein dairy fed a post-partum diet plus fat prill containing 85% palmitic acid (control group) or plus Carneon 20 Rumin-pro (RLC group).

**Fatty acids**	**Experimental groups**		**SEM**	***p-*value**
	**Control**	**RLC**		
C4:0	6.62	7.55	1.016	ns
C6:0	3.52	4.40	0.642	ns
C8:0	1.83	2.18	0.327	ns
C10:0	3.07	3.33	0.484	ns
C12:0	2.75	2.53	0.273	ns
C14:0	7.70	7.07	0.384	ns
C14:1 *cis-9*	1.22	1.07	0.076	^*^
C15:0	0.85	0.70	0.043	^*^
15:1	0.20	0.26	0.027	ns
C16:0	23.64	23.75	0.685	ns
C16:1 *cis-9*	2.68	2.35	0.122	^*^
C17:0	0.65	0.66	0.031	ns
C17:1	0.32	0.33	0.027	ns
C18:0	12.38	12.09	0.637	ns
C18:1 *trans-9, trans-11*	0.94	1.40	0.133	^*^
C18:1 *cis-9*	24.92	24.67	1.324	ns
C18:2	0.90	0.60	0.138	^*^
C18:2 *cis-9*	4.38	3.59	0.487	ns
C20:0	0.70	0.73	0.151	ns
C18:3n-3	0.45	0.48	0.059	ns
C20: >1; n_3_,n_6_	0.31	0.24	0.042	ns
Saturated fatty acids	63.70	65.01	1.878	ns
Monounsaturated fatty acids	30.28	30.08	1.565	ns
Polyunsaturated fatty acids	6.02	4.90	0.542	ns
C18:2 cis-9/C15	27.34	26.01	2.21	ns

* *p < 0.05; ns: p > 0.05*.

### Blood Metabolites

Pre- and post-partum blood serum metabolites are presented in Table 5. Results of the current study showed that the effect of the experimental group on cholesterol (mg/dL), HDL (mg/dL), LDL (mg/dL), haptoglobin (mg/dL), SGPT (U/l), SGOT (U/l), and NEFA (mmol/l) was significant (*p* < 0.05). Animals in the RLC group had a lower blood cholesterol concentration compared with those of the control group (99.4 and 82.6 mg/dl for the control and RLC groups, respectively). Pre- and post-partum dietary inclusion of the rumen-protected L-carnitine source decreased both LDL and HDL concentrations in peripheral blood of post-partum cows in the present study. The concentrations of cholesterol, HDL, and LDL decreased at 7 days before calving and eventually increased from 2 weeks after calving. In the present experiment, rumen-protected L-carnitine supplementation hardly influenced the albumin concentration during the transition period (*p* > 0.05). The serum albumin level in cows of both experimental groups increased by 8% after calving, whereas the urea concentration in the animals hardly changed compared with the pre-partum period. Results from this work showed that cows in the RLC group had significantly (*p* < 0.05) lower blood haptoglobin concentrations at 7 and 14 days after calving than the control group (22.8 and 11.1 mg/dl for the control and RLC groups, respectively). Both serum glucose and calcium concentrations declined as parturition approached and started to increase from 21 days post-calving. There was no significant effect (*p* > 0.05) of the experimental group or group and day interactions on blood calcium or glucose concentration. Data regarding the concentration of blood triglycerides indicated that there were no significant differences between the groups. The blood NEFA concentration was evidently decreased in cows supplemented with rumen-protected L-carnitine during the periparturient period (*p* < 0.05). Animals in the control group had a 20% higher NEFA concentration compared with those levels in the RLC group. Nevertheless, dietary rumen-protected L-carnitine hardly changed the BHB level in animals in comparison with control (*p* > 0.05). Generally, both blood NEFA and BHB concentrations were increased before calving (50 and 30% for NEFA and BHB, respectively) and decreased from 14 DIM onward. Circulating concentrations of both blood enzymes, i.e., SGPT and SGOT, increased from 1 week before calving. Moreover, animals in the RLC group had lower concentrations of both enzymes than the control group (*p* < 0.05).

**Table 5:** Concentration of blood metabolites in Holstein dairy cows during the transition period fed diets plus fat prill (control group) or Carneon 20 Rumin-Pro (RLC group).

**Items**	**Experimental groups**										**SEM**	***p*** **-value**		
	**Control**					**RLC**						**Group**	**Time**	Group^*^Time
	**−14 days**	**−7 days**	**+7 days**	**+14 days**	**+21 days**	**−14 days**	**−7 days**	**+7 days**	**+14 days**	**+21 days**		
Glucose (mg/dL)	56.8	52.1	48.8	42.2	50.7	54.1	49.4	47.2	45.7	49.2	0.77	ns	^*^	ns
Urea (mg/dL)	38.0	35.3	40.5	39.8	43.2	39.0	32.0	40.5	40.2	39.7	0.62	ns	ns	ns
Albumin (g/dL)	3.94	3.95	3.92	4.00	4.14	3.80	3.81	3.82	3.82	3.99	0.04	ns	^*^	ns
Cholesterol (mg/dL)	104	102	77.6	89.9	125	92.3	79.0	65.8	76.6	99.6	2.40	^*^	^*^	ns
HDL (mg/dL)	81.8	82.4	68.7	73.7	90.6	74.5	67.1	57.5	61.0	78.5	1.57	^*^	^*^	ns
LDL (mg/dL)	12.6	9.60	7.60	11.5	15.9	9.80	8.30	5.50	8.60	12.7	0.50	^*^	^*^	ns
Triglyceride (mg/dL)	25.1	27.9	12.0	10.4	12.3	27.2	27.0	12.7	10.2	8.2	1.11	ns	^*^	ns
Haptoglobin (mg/dL)	-	-	19.5	26.1	-	-	-	10.9	11.3	-	2.10	^*^	ns	ns
Calcium (mg/dL)	8.75	8.69	7.98	8.67	9.27	8.56	8.43	7.90	8.49	9.11	0.15	ns	^*^	ns
SGPT (U/l)	18.5	16.8	16.6	17.1	20.1	14.9	13.4	12.5	14.9	18.5	0.56	^*^	^*^	ns
SGOT (U/L)	75.3	80.5	103	103	107	79.2	72.9	90.6	99.8	105	2.50	^*^	^*^	ns
NEFA (mmol/L)	-	0.43	1.15	1.05	0.75	-	0.33	0.83	0.89	0.77	0.05	^*^	^*^	ns
BHBA (mmol/L)	-	0.53	0.67	1.01	0.65	-	0.53	0.77	0.85	0.75	0.04	ns	^*^	ns

*d: day relative to calving;*

* *p < 0.05; ns: p > 0.05; when the difference between means is >2 times the SEM., it is considered as significant (p < 0.05)*.

### mRNA Expression

**Figure 2:**
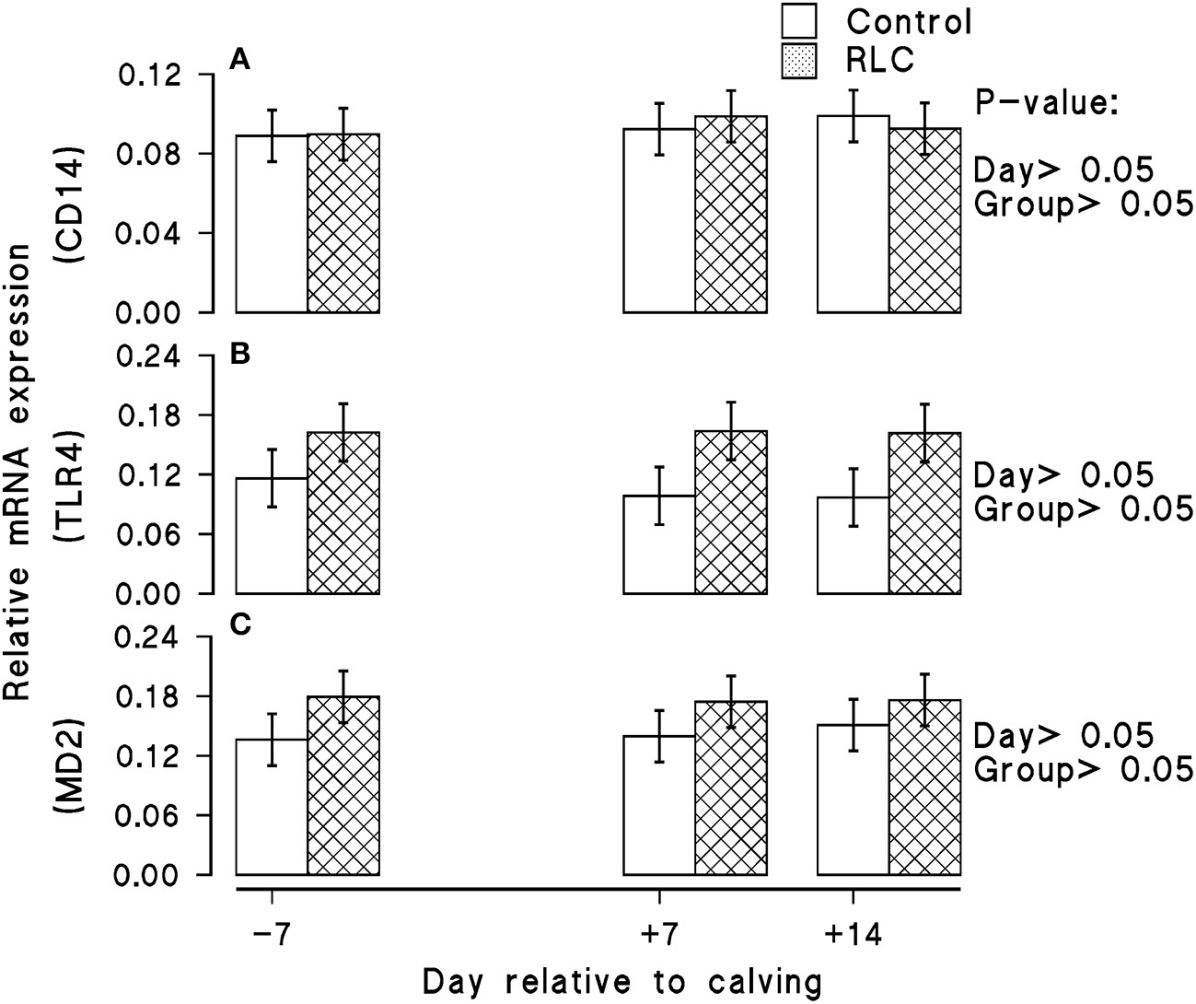
Effects of supplementing multiparous Holstein cows during the transition period with rumen-protected L-carnitine (RLC; Carneon 20 Rumin-Pro, Kaesler Nutrition GmbH, Cuxhaven, Germany) on the relative mRNA expression of CD14 **(A)**, TLR4 **(B)**, and MD2 **(C)** genes. Values are means, with standard errors of means (*n* = 10/group) represented by vertical bars. The group fed rumen-protected L-carnitine depicted as RLC.

Effects of the dietary inclusion of rumen-protected L-carnitine on mRNA abundance of CD14, TLR4, and MD2 of early lactating Holstein dairy cows is shown in Figures 2A–C, respectively. Results of the current work indicated that supplementing the transition diet with rumen-protected L-carnitine hardly showed any significant impact on the mRNA abundance of TLR4, CD14, and MD2 (*p* > 0.05).

### Animal Behavior

**Figure 3:**
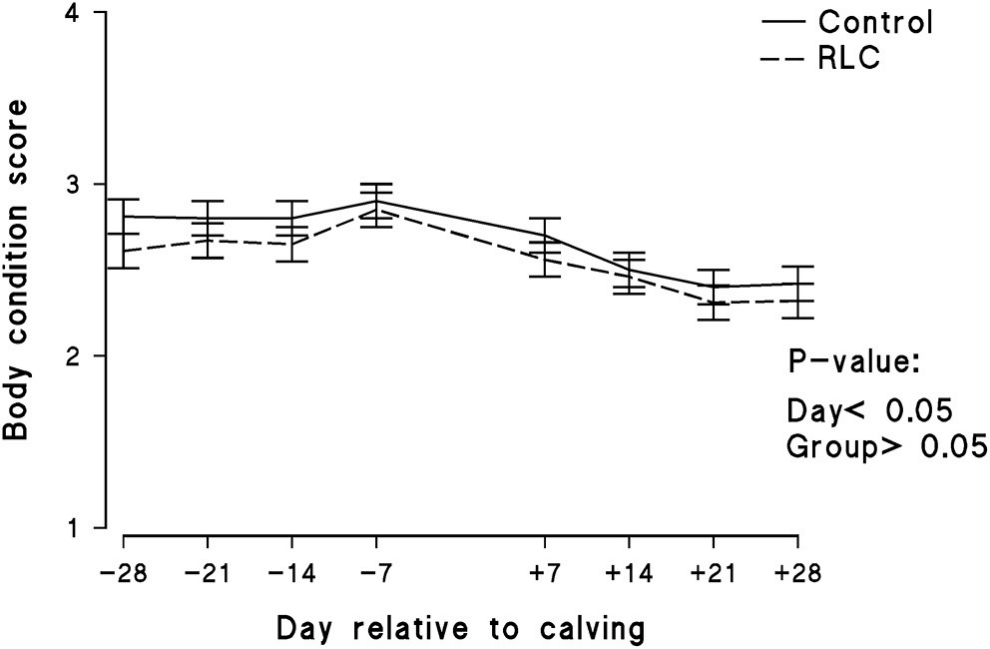
Effects of supplementing multiparous Holstein cows during the transition period with rumen-protected L-carnitine (Carneon 20 Rumin-Pro, Kaesler Nutrition GmbH, Cuxhaven, Germany) on the body condition score of the animals. Values are means, with standard errors of means represented (*n* = 16/group) by vertical bars. The group fed rumen-protected L-carnitine depicted as RLC.

**Figure 4:**
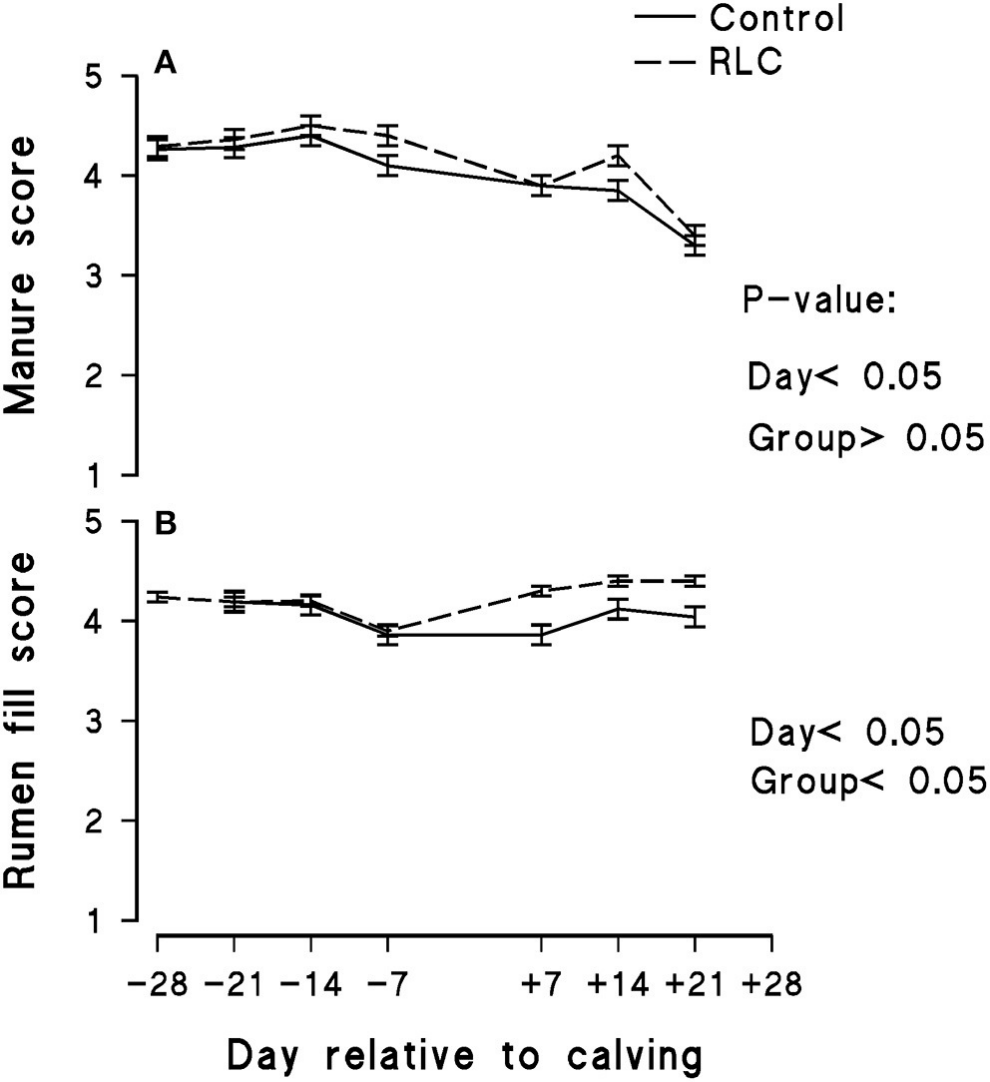
Effects of supplementing multiparous Holstein cows during the transition period with rumen-protected L-carnitine (Carneon 20 Rumin-Pro, Kaesler Nutrition GmbH, Cuxhaven, Germany) on manure score **(A)** and rumen fill score **(B)**. Values are means, with standard errors of means (*n* = 16/group) represented by vertical bars. The group fed rumen-protected L-carnitine depicted as RLC.

**Figure 5:**
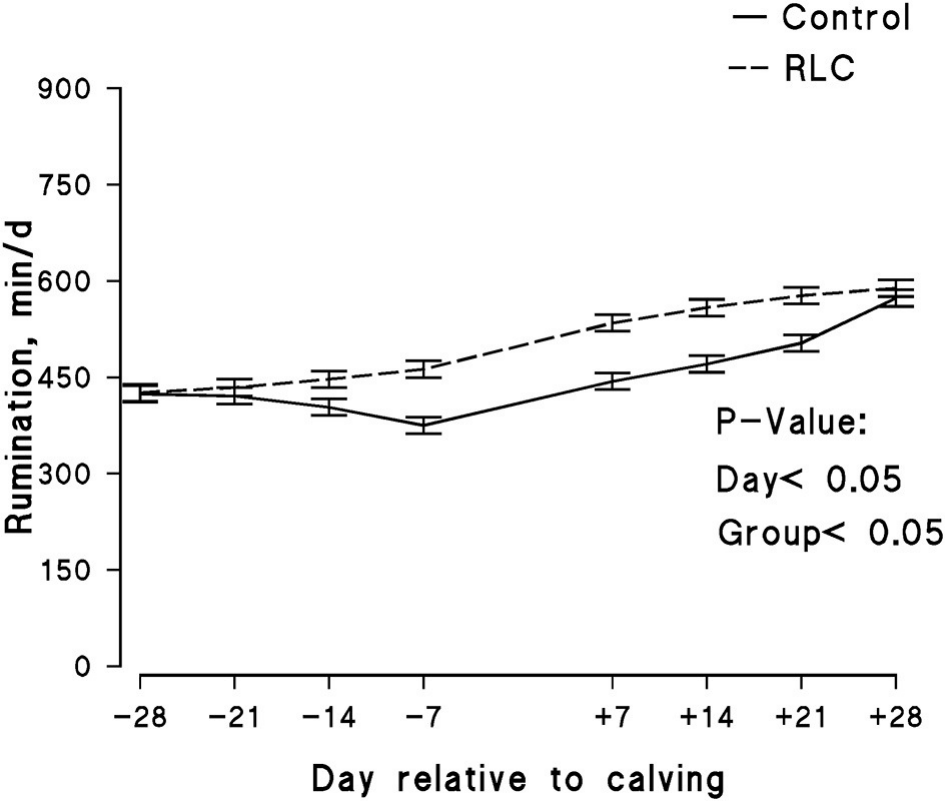
Effects of supplementing multiparous Holstein cows during the transition period with rumen-protected L-carnitine (Carneon 20 Rumin-Pro, Kaesler Nutrition GmbH, Cuxhaven, Germany) on rumination activity. Values are means, with standard errors of means (*n* = 16/group) represented by vertical bars. The group fed rumen-protected L-carnitine depicted as RLC.

The experimental group effect on BCS of the animals throughout the study is presented in Figure 3. L-Carnitine supplementation did not evidently affect the BCS of the animals. The initial BCSs of animals in both groups were similar; however, it decreased with the increase in days post-calving. The data of manure score and rumen fill score are shown in Figures 4A,B, respectively. The manure score was unaffected by the experimental groups. The rumen fill score was significantly (*p* < 0.05) influenced by the experimental group and days relative to calving. Animals in the RLC group had a higher rumen fill score in comparison with the control. The rumination activity for each experimental group is presented in Figure 5. There was a significant difference (*p* < 0.05) among the cows allocated in the experimental groups, in which animals in the RLC group demonstrated 11% higher rumination time compared with their counterparts in the control group.

## Discussion

Results from the present experiment underline the ability of L-carnitine, in a protected form, to support the production responses, enhancing the liver metabolism and modulating the health biomarkers of high-producing dairy cows during the periparturient period. This study was able to show significant changes in particular cow behavior indices, which can be used for identifying cows with risk of illness (46). Taken together, data generated from this experiment could help develop more detailed feeding management, i.e., including particular feed additives, to ensure dairy cattle to overcome the transition from parturition to lactation with less noticeable damage to their entire lactation performance and longevity.

### Productive Performance of the Animals

Our results showed that the animals in the RLC group had better productive responses. It has been demonstrated that energy intake during early lactation is insufficient to meet the animal needs for milk synthesis. Indeed, cows very often enter into a negative energy balance where their successful lactation would be effectively disturbed (47). Therefore, dairy cattle during the transition from gestation to lactation requires substantial nutrients to shift their situation (48). Previous studies claimed that increasing the energy availability of the transition diet through L-carnitine supplementation may have some benefits (26, 49), allowing the animals to adapt and decrease fatty acid mobilization from adipose tissue, eventually being less prone to lipid-related metabolic disorders (50). Hence, following parturition, this resulted in cows showing a better milk production and composition responses as those observed in the present work. Moreover, the findings of the current study suggest that upon dietary inclusion of an L-carnitine source, resisting ruminal degradation, to both pre- and post-partum diets, cows produce more milk components. It seems that a more favorable metabolic situation by including rumen-protected L-carnitine in the transition diet may decrease the negative physiological situation of this period (48). Therefore, effects of the rumen-protected L-carnitine supplementation to the transition diets cause an increase to the uptake of energy and nutrients for milk yield components. The outcome from our investigation indicated an obvious lower milk SCC in dairy cattle fed with L-carnitine. These results are not in line with previous studies, in which dietary supplementation of L-carnitine did not show any significant changes in SCC parameter (26). Scholz et al. (51) merely observed numerical lower SCC in dairy cows fed rumen-protected L-carnitine in comparison with control. Regular SCC observation has been globally recognized as an optimal index for measuring inter-mammary infection and milk quality (52). This parameter along with udder health monitoring programs has been quite advantageous on individual cows as well as the entire herd (53). Current data indicated that cows receiving rumen-protected L-carnitine, i.e., increasing the post-ruminal L-carnitine availability, could be less prone to develop mammary disorders in early lactation period. This is quite important for the productivity and longevity of the animals in the subsequent lactation.

Data from our experiment indicated that dietary supplementation with rumen-protected L-carnitine was able to merely regulate few milk fatty acid concentrations during the early lactation period. In dairy cows, the short- and medium-chain fatty acids (C4:0–C14:0) are synthesized in the mammary gland. Overall, short- and medium-chain fatty acids were numerically higher in animals in the RLC group. The only difference between the C14:1 cis-9 concentrations was significant. Buitenhuis et al. (54) evaluated the effect of microbiome on milk fatty acid composition and reported the heritability and microbiability for each trait. They showed that, in general, the heritability was relatively high for all milk fatty acids [ranging from 0.69 (C14:1 cis-9) to 0.11 (C18:1 trans-11; C18:1 cis-9)]. Therefore, the difference in C14:1 cis-9 obtained in the present study might be explained by the different rumen microbiomes. During the negative energy balance period, by increasing the energy demand for milk production, body fat is mobilized and transported as NEFA to several organs, particularly to the liver (15, 55). Excessive amounts of NEFA [particularly rich in long-chain fatty acids, e.g., C18:1 cis-9 and C18:0 (56)], which is released during body fat mobilization, have a potential to transfer to the milk, resulting in their elevated concentrations in the milk fat. It has been proposed that these fatty acids in milk fat were identified as valuable early warning biomarkers for health status during transition period (57). Jorjong et al. (58) assessed the potential of milk fatty acids as biomarkers to predict the health status of the early-lactating dairy cows. The authors claimed that the milk fat C18:1 cis-9/C15:0 ratio may be a useful factor for the diagnosis of hyperketonemia in early-lactating dairy cows. Therefore, based on the data reported by Jorjong et al. (58), the ratio of C18:1 cis-9/C15 between 34 and 45 seems to be a valuable threshold in the early-lactating dairy cows. In the current study, the ratio of C18:1 cis-9/C15:0 for each experimental group was below 40 (Table 4). This shows that the animals enrolled in the current study were not generally in a critical health status.

In the current study, we used similar fat concentrations (with the same sources) in the experimental diets. We even used very similar concentrations of palmitic acid in the diet. Dietary fat enhances the supply of fatty acids to the mammary gland, which results in a lower proportion of *de novo* synthesized, saturated short- and medium-chain fatty acids in milk fat and a higher proportion of long-chain fatty acids (59). On the other hand, any differences in the supply of fermentable carbohydrate lead to altered production of acetate in the rumen, as a precursor of mammary *de novo* fatty acid production. Therefore, it has been proposed that fatty acids with <16 C originated from *de novo* synthesis and those >16 C were preformed fatty acids taken up by the mammary gland, and 16:0 and 16:1 fatty acids come from both *de novo* and preformed sources (59, 60). However, Dewhurst et al. (61) showed that actual milk yields of C15:0 and C17:0 exceeded the duodenal flow of these fatty acids. They suggested that there is a possibility of some *de novo* synthesis within animal tissue or transfer of these fatty acids (mobilized from adipose tissue) to the mammary gland. All of the above information clearly stated that when we used similar dietary carbohydrates (i.e., similar fermentation pattern) and fat supplements, as well as when observing similar BCS (i.e., same fat mobilization), it should be expected to observe relatively similar milk fatty acid profiles between the experimental groups.

### Metabolic and Health State of the Animals

Data from the current study revealed that dietary inclusion of L-carnitine resulted in lower circulating concentrations of cholesterol, HDL, and LDL. Typically, the cholesterol concentration in dairy cows declines close to parturition and starts to gradually increase post-parturition (62), following the pattern of changes in animals' feed intake during this period (63). Previous studies have pinpointed higher risk of post-partum diseases associated with higher cholesterol concentrations pre-partum (62). The total cholesterol level in the blood has been also attributed to the changes in serum lipoprotein concentrations during lactation (64). Therefore, lower concentrations of LDL and HDL in dairy cows in the RLC group were expected. Stefanska et al. (37) reported higher concentrations of HDL in non-healthy cows compared with the healthy group (78.16 vs. 68.32 mg/dl). They proposed that higher concentrations of blood HDL in non-healthy cows may be a protective mechanism against endotoxemia. Our data regarding the blood HDL concentration confirmed those findings. More recently, Jukema et al. (65) indicated that disorders in lipid metabolism initiate an inflammatory and immune-mediated response, in which the concentration of blood LDL was increased. Hence, they suggested that blood LDL cholesterol has strong potential to induce inflammation in animals. The lower blood LDL concentration in cows fed the rumen-protected L-carnitine may explain a much better health status in these animals as observed in the current experiment.

In the present work, we have determined the circulating concentrations of two acute phase proteins (APP), i.e., albumin and haptoglobin. The liver is vital for an optimal immune response as it will redirect the priority from metabolism to defense during an incidence of inflammation in animals. This change is known as APP response, which depicts the reduced synthesis of necessary proteins for normal liver metabolism, e.g., albumin, retinol-binding protein, paraoxonase, and increased synthesis of proteins, which are involved in immune and detoxification response (66). Positive APP play an important role in pathogen elimination, removal of toxic substances, and maintenance of a balanced inflammatory response (67). Haptoglobins are among the positive APP, in which their blood levels increase as a result of an inflammation stage in animals (68). Stefanska et al. (37) reported a lower blood concentration of haptoglobin in healthy vs. acidotic cows (470.19 vs. 516.85 ng/ml). A previous experiment revealed that elevated concentrations of blood haptoglobin from 2 to 8 days post-partum were associated with enhanced innate immune responses (69). These findings provide evidence that the blood haptoglobin concentration is associated with both systemic inflammatory responses and liver inflammation. Post-partum blood concentrations of haptoglobin >1.1 g/l were associated with a 947-kg decrease in 305-day mature equivalent milk yield (70). Inflammatory response during the periparturient period has been characterized with an increase in the production of positive APP and a concomitant decrease in the production of negative APP, e.g., albumin (71). The lower serum haptoglobin concentration in cows in the RLC group may indicate a better health status of the animals. This in part could be the reason that animals in this group produce higher fat- and energy-corrected milk during 4 weeks after calving. Nevertheless, we did not observe any significant difference in the serum albumin level between the groups. Others also did not find a clear difference in blood albumin concentration of animals treated with anti-inflammatory drugs from −7 until +35 days relative to calving, while haptoglobin levels were clearly decreased upon treatment (72).

Although the blood concentrations of triglyceride and BHB did not significantly differ between the groups, dietary L-carnitine supplementation showed an evident effect to decrease the circulatory NEFA concentration. In dairy cows when there is an energy deficiency, a mobilization of body fat reserves occurs and, thus, the concentration of NEFA increases in blood (21). On the other hand, an efficient utilization of NEFA depends on an adequate L-carnitine availability for fatty acid transfer into the mitochondrial matrix as the site of their oxidation (73). Insufficient L-carnitine availability at times of an increased energy requirement, such as early lactating status, could alter the liver metabolism of lipids. Carlson et al. (74) demonstrated that carnitine modulates nutrient metabolism in dairy cows. They indicated that carnitine supplementation of 50 and 100 g/day had more potent effects on lipid metabolism as a result of enhanced capacity for hepatic long-chain fatty acid β-oxidation. In their experiment, the marked increases in hepatic carnitine concentrations confirm that exogenous carnitine is readily taken up by the liver. This idea has also been previously demonstrated in mid-lactation dairy cows (73). Therefore, an insufficient L-carnitine supply to the liver was proposed as a limiting factor for fatty acid metabolism (75). Besides, it has been shown in dairy cows that many LPS-induced metabolic challenges are related to the energy metabolism, in which L-carnitine is involved (76). The authors showed that an intravenous LPS injection followed by an increase in blood tumor necrosis factor-α was accompanied with a rise in blood NEFA concentration.

Metabolic alterations in the liver during the transition period are one of the key points in dairy cow performance (21). As seen in our results, an evident decline of the liver enzymes during the periparturient period in rumen-protected L-carnitine-supplemented animals seems to improve their productive responses in the subsequent lactation. Recently, a number of blood metabolites were used to monitor clinical or subclinical signs of metabolic disorders in high-yielding cows around parturition (1). The blood elevation of SGPT and SGOT may indicate an accumulation of NEFA transported from blood to hepatocytes (77). Higher concentrations of blood SGOT can also imply damages in organs other than the liver, as SGOT exists in the muscle, kidney, intestine, and brain. West (78) proposed that a rise in blood SGOT shortly after calving might indicate a muscle damage in the animals. Therefore, metabolic alterations in liver function may be an important point in early lactating cows. Olagaray et al. (49) reported that fatty liver is a metabolic disease that occurs during the first few weeks of lactation and affects up to 50% of dairy cows. Higher incidence of fatty liver accompanies with a decline in the concentration of free carnitine. Dietary administration of L-carnitine during the transition period was effective at increasing hepatic carnitine concentrations, with a subsequent decrease in total liver lipid content (79). This will in turn help the liver to reduce the hepatocyte damages, moderate its metabolic function, and enhance the health status and productive performance of the animal.

### mRNA Abundance of TLR4, CD14, and MD2

Results from the current work indicated that the expression of genes involved in bacterial LPS recognition was not evidently regulated upon L-carnitine supplementation. During states of inflammation and inflammatory disease, the expression of TLR4 and associated signaling proteins could increase and facilitate receptor-mediated endocytosis of LPS (80, 81). Results of the current study are not in agreement with previous works, where higher transcription levels of TLR4 and CD14 were noticed in early-lactating dairy cow with puerperal diseases (82, 83). Interaction of TLR4/CD14/MD-2 with the bacterial endotoxin triggers the expression of inflammatory biomarkers such as cytokines, antimicrobial peptides, and chemokines. A plausible reason for the current outcome would be that animals during the course of this experiment were not suffering from clinical inflammatory diseases associated with the periparturient period.

### Behavioral Parameters of the Animals

We did not observe any evident differences of manure scores, yet the rumen fill scores were significantly affected by the addition of L-carnitine (Figures 4A,B). The rumen fill score is related to the DMI, especially the proportion of fiber in the diet (43). Kawashima et al. (84) showed that the rumen fill score is also associated with energy status in dairy cows. They concluded that the rumen fill score during the transition period might indicate the real-time feed intake based on its correlation with serum total cholesterol levels. Consequently, it might be used as a practical indicator to describe the metabolic health status in dairy cows. In the present study, all cows had very good rumen fill scores during the pre- and post-partum periods. Nevertheless, addition of rumen-protected L-carnitine in transition diets was able to enhance this parameter in the animals.

It has been previously reported that early-lactation cows would produce much more milk per unit of dry matter consumed and per minute of rumination resultant from that feed consumed (85). In ruminants, rumination is a natural behavior and mostly influenced by the physically effective fiber (86), which would increase the surface area of the feed particle. In addition, rumination stimulates saliva production to help buffer the rumen and facilitates the passage of dry matter from the rumen to the intestine. Therefore, as the passage rate increases, DMI increases in dairy cows (85), eventually improving the productive responses of the animals. The time that cows spend chewing might be a valuable management tool for detecting health problems and optimizing the herd heath status (40), as monitoring rumination time is easier than monitoring feed intake. Kaufman et al. (85) monitored the relationship between health status of dairy cows with rumination time, milk yield, milk fat, and protein content. They concluded that rumination time was positively associated with health status and milk yield in early-lactation dairy cows across all parities. It was concluded that healthy animals had better productive responses compared with unhealthy cows across all lactations. Besides, early-lactating cows are more challenging in the view of metabolic stress and are more susceptible to health disorders that cause significant production losses. Stangaferro et al. (87) evaluate the rumination time to score the health of cows from 21 days before expected calving until 80 days post-partum. The rumination time of cows was lower in clinical diagnosis, depending on the disorder, compared with the healthy cows. Taken together, higher rumination time in cows of the RLC group in the present work would be an indicator of the better health situation of the animals.

## Conclusion

Altogether, results of the present experiment provide evidence that dietary inclusion of rumen-protected L-carnitine during the transition period could improve the productivity of high-producing dairy cows early post-partum. This can be to some extent explained by the ability of L-carnitine to modulate metabolic indicators as indexed in the energy metabolism and liver functionality. This experiment proves that eating and ruminating of transition dairy cows will be positively affected by the dietary inclusion of L-carnitine. Although the overall health of the animals enrolled in the current study was relatively good, addition of L-carnitine seems to even enhance the health status of the animals. However, further research is warranted to gain a deeper understanding on the impact of pre- and post-partum dietary L-carnitine on various pro- and anti-inflammatory as well as oxidative status biomarkers in dairy cows. Another compelling idea would be to investigate the *in-utero* effect of feeding L-carnitine during the periparturient period, thereby determining the potent impact of this natural molecule on the offspring performance and health.

## Data Availability Statement

The original contributions presented in the study are included in the article/Supplementary Material, further inquiries can be directed to the corresponding author.

## Ethics Statement

The animal study was reviewed and approved by Institutional Animal Care Committee, Ferdowsi University of Mashhad.

## Author Contributions

MDM, HK, and SDM conceived and designed the study and wrote the paper with a critical review by all authors. HK conducted the experiment. AJ conducted the qPCR analysis. All authors reviewed and approved the final manuscript.

## Funding

Partial support for the conduct of this research was provided by Kaesler Nutrition GmbH, Cuxhaven, Germany. Kaesler Nutrition GmbH had a role in the study design and provided financial support to cover the costs of sample analysis and data collection.

## Conflict of Interest

SDM is employed by Kaesler Nutrition GmbH. The remaining authors declare that the research was conducted in the absence of any commercial or financial relationships that could be construed as a potential conflict of interest.

## Publisher's Note

All claims expressed in this article are solely those of the authors and do not necessarily represent those of their affiliated organizations, or those of the publisher, the editors and the reviewers. Any product that may be evaluated in this article, or claim that may be made by its manufacturer, is not guaranteed or endorsed by the publisher.
